# Case Report: A Case of Eyelid Myoclonic Status With Tonic–Clonic Seizure and Literature Review

**DOI:** 10.3389/fped.2021.671732

**Published:** 2021-04-22

**Authors:** Yujun Yuan, Fenghua Yang, Liang Huo, Yuying Fan, Xueyan Liu, Qiong Wu, Hua Wang

**Affiliations:** Department of Pediatrics, Shengjing Hospital of China Medical University, Shenyang, China

**Keywords:** eyelid myoclonus, Jeavons syndrome, status epilepticus, photosensitiveness, eye closure sensitivity

## Abstract

Eyelid myoclonus with or without absence epilepsy is a rare and usually misdiagnosed disease in the neurology department. It is an idiopathic general epileptic syndrome, the onset period is 6–8 years, and is more common in girls. It is characterized by rapid abnormal eye blinking, accompanied by upward rolling of the eye and slight backward movement of the head, with eye closure sensitivity and photosensitivity. The seizure is frequent and short, dozens or even hundreds of times a day; a small number of patients may have eyelid myoclonus status. We report a patient who visits the hospital for the first time with eyelid myoclonic problem; the patient continued to wink the eyes, eye rolled up, and backward movement of the head, accompanied by impairment of consciousness. Video electroencephalography (VEEG) suggests continued spike slow-wave, polyspike slow-wave. After the patient had 2, 4, 6, 8, 10, 12, and 14 Hz of intermittent photic stimulation (IPS), her seizures and epileptic discharges reduced or stopped. Seven min after giving stimulation at 20 Hz, the child developed an occipital-initiated tonic–clonic seizure, which demonstrated that after sufficient IPS stimulation, the occiput cortex became excited and initiated a brain network, leading to diffuse brain discharge and tonic–clonic seizures. At 1 h after onset, the child developed a nonconvulsive state, with impairment of consciousness despite no eyelid myoclonic movements, and VEEG suggested a large number of epileptic discharges. After 10 min of administrating midazolam, the patient's EEG immediately became normal, and the patient regained consciousness. Therefore, this paper presents an eyelid myoclonus status patient with occipital origin seizure, we recorded the whole course of the disease and the treatment effect, and reviewed the literature accordingly.

## Introduction

Eyelid myoclonus with absence (EMA) or Jeavons syndrome is a rare idiopathic generalized epilepsy (IGE) that occurs in childhood. The symptomatic features of eyelid myoclonus were first reported by Radovici et al. (1) more than 80 years ago. The study of epileptic discharges and seizures caused by closing the eyes was reported in 1955 (2). This epileptic syndrome was first fully described by Jeavons in 1977. It is characterized by eyelid myoclonus, with or without absence of seizures, eyelid closure induces epileptic discharge and/or seizures, and photosensitivity. Subsequently, EMA was again confirmed as an independent individual in IGE. Eyelid myoclonus is a hallmark of Jeavons syndrome. Usually, it is associated with repeated myoclonic jerks of the eyelids, eyeballs roll upward, and the head may move slightly backward. When accompanied by impairment of consciousness, it is named eyelid myoclonus with absence. Eyelid myoclonic status may be spontaneous or light stimulated. It consists of recurrent and discontinuous seizures of eyelid myoclonus, accompanied by mild absence (3). Many patients with IGE have visual precursors associated with photosensitive and focal epilepsy, EMA is common in IGE patients with photosensitivity. The occipital cortex can be activated by eye closure and IPS, and this excitability may spread to the brain stem, resulting in eyelid myoclonus. As the intensity of the photic stimulus increases, the excitability can be projected into the central frontal cortex via transcortical and thalamocortical pathways, and then generalized seizures and absence seizures may occur (4). However, focal epileptic discharge is also present in patients with IGE. Gungor-Tuncer et al. had demonstrated that epileptic discharges of focal origin can still occur in some IGE patients. The interictal focal discharge independent of localization in some patients suggests that there may be an overlap between focal epilepsy and generalized epilepsy (5). Eyelid myoclonus has some overlap with other seizures, especially photosensitive epilepsy, and reflex epilepsy; therefore, the differential diagnosis of EMA is very important. Patients with eyelid myoclonic status are even less common in clinical practice. Giuliano et al. (6) found that 17.6% of patients with eyelid myoclonus had epileptic status. Striano (1) also reported that one in five patients might experience eyelid myoclonus status. We input the keywords “eyelid myoclonic status” when searching in PubMed. There were only three reported cases (7–9). Valproate is widely recognized as the most effective treatment for photosensitive epilepsies (10). Therefore, we present a very rare case of eyelid myoclonus status followed by an occipital origin of tonic–clonic seizure with the clinical manifestations, electroencephalogram (EEG) characteristics, differential diagnosis, and therapeutic effect in detail, and reviewed the literature.

## Case Report

**Figure 1:**
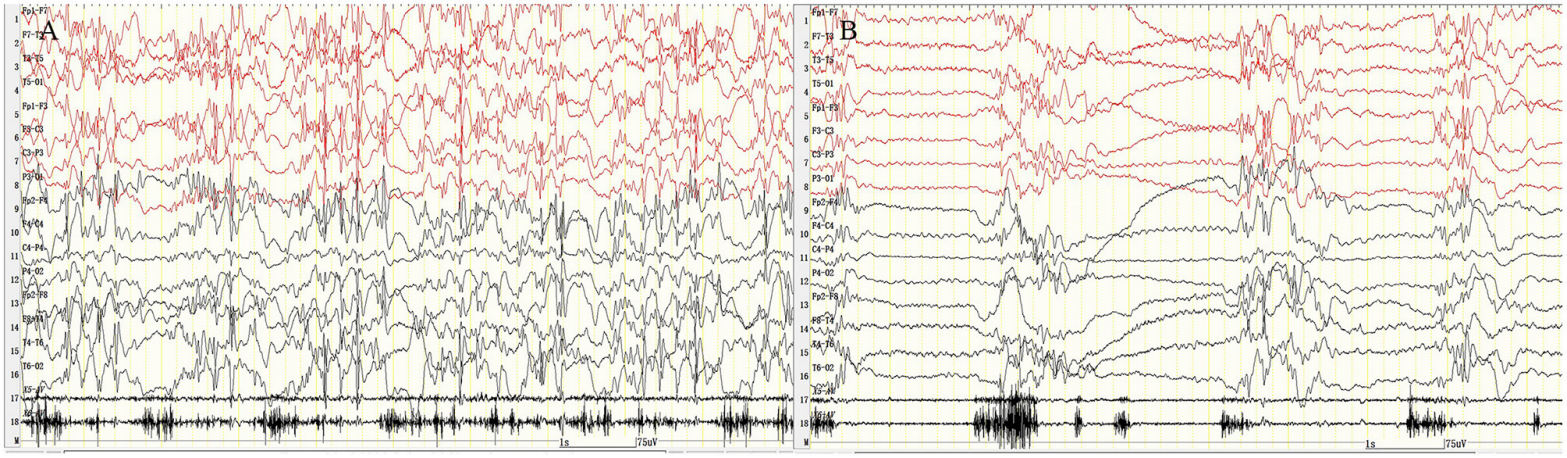
Eyelid myoclonic status. The patient was continuously blinking with upward rolling of the eyeball and backward movement of the head; VEEG: sustained and extensive 3- to 6-Hz high-amplitude spike–slow wave, polyspike–slow wave discharge. **(A)** Fast blinking movement. **(B)** Slow blinking movement. X5, outer eyelid; X6, upper eyelid. SEN: 15 μV; HF: 70; TC: 0.3.

**Figure 2:**
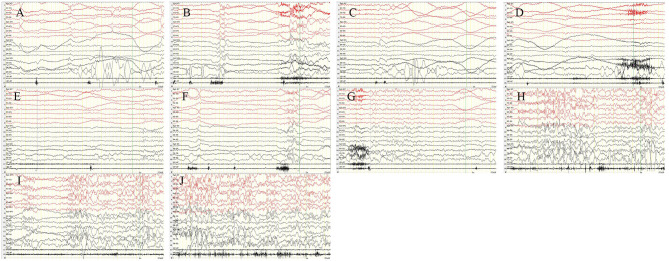
Intermittent photic stimulation (IPS). After the patient had 2 Hz **(A)**, 4 Hz **(B)**, 6 Hz **(C)**, 8 Hz **(D)**, 10 Hz **(E)**, 12 Hz **(F)**, and 14 Hz **(G)**, the epileptic discharges reduced or stopped. But after 16 Hz **(H)**, 18 Hz **(I)**, and 20 Hz **(J)**, the epileptic discharges increased and blinking started again. X5, outer eyelid; X6, upper eyelid. SEN: 15 μV; HF: 70; TC: 0.3.

**Figure 3:**
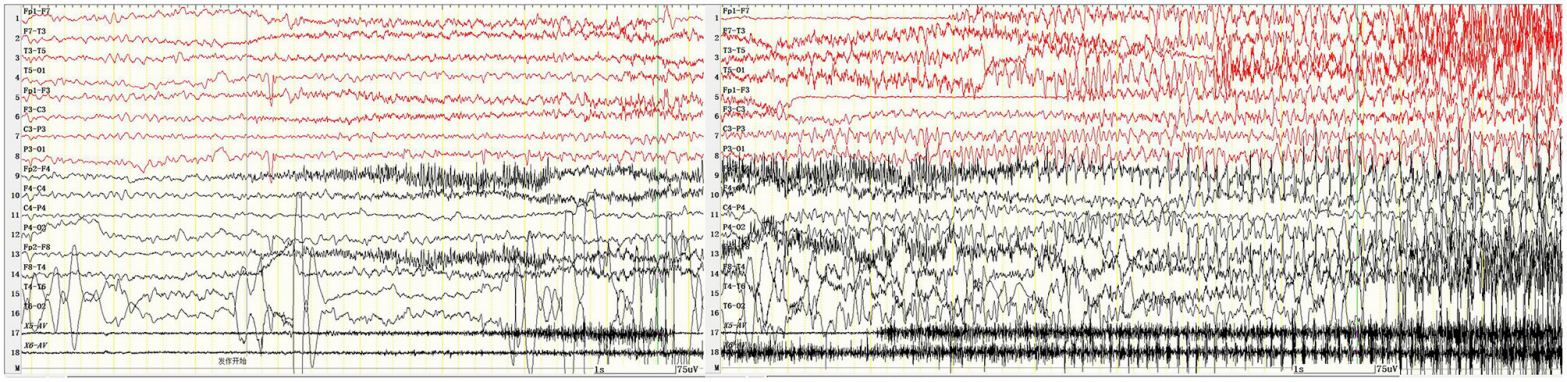
Tonic clonic seizures beginning in the occipital region 7 min after IPS. X5, outer eyelid; X6, upper eyelid. SEN: 15 μV; HF: 70; TC: 0.03.

**Figure 4:**
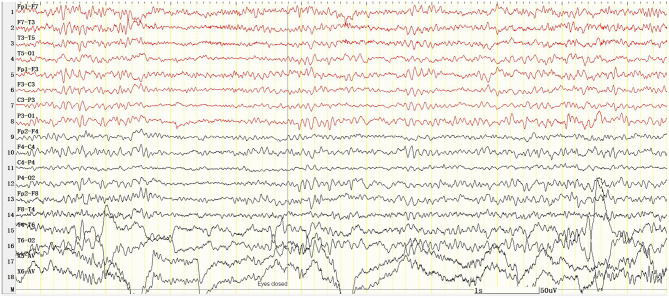
There was no epileptic discharge in VEEG 10 min after the administration of midazolam, and the background rhythm was about 8 Hz. X5, outer eyelid; X6, upper eyelid. SEN: 10 μV; HF: 70; TC: 0.1.

A 12-year-old girl was admitted to the hospital (*Department of Pediatrics, Shengjing Hospital of China Medical University, Shenyang, China*) with the main complaint of “frequent blinking for 4 h.” Her main manifestations were continuous blinking, upward rolling of the eyeball, backward movement of the head, and accompanied by decreased consciousness. Physical examination showed poor condition, continuous eyelid blinking with unclear consciousness; neck strength (–), Babinski sign (–), limb muscle tone was normal, and muscle strength grade V. Physical examination of other nervous systems was normal. After hospitalization, video electroencephalography (VEEG) was performed immediately. It showed persistent spike–wave, polyspike–wave discharge (Figure 1), especially when the eyes were closed. At the same time, the patient had low response ability, slow speech, and slow action. However, she could answer the questions accurately. It suggested that the patient had an eyelid myoclonus status accompanied by absence. The patient had an IPS test; when at 2, 4, 6, 8, 10, 12, and 14 Hz frequency, the seizures and epileptic discharges reduced or stopped (Figure 2). However, after the patient had 16, 18, and 20 Hz, the epileptic discharges increased and blinking started again. Seven min after 20 Hz, the VEEG found a tonic–clonic seizure starting from the occipital region (Figure 3). That indicated that the patient had photosensitivity. After a seizure, her eyelid myoclonus symptoms disappeared, and simultaneously, EEG discharge decreased significantly. However, the girl was heavily drowsy, and slow-wave increased. 30 minutes after onset, the VEEG showed that epileptic discharges increased again and after 1 hour of onset, the epileptic discharge increased to the initial state-continuous discharge. We hypothesized the girl was experiencing a non-convulsive status; therefore, we gave her midazolam 5 mg via intravenous injection immediately and then 2 ml/h (0.05 mg/kg/h) of intravenous fluid. After 10 min, the EEG showed that the epileptic discharge disappeared and started recovering to normal state (Figure 4). Besides, the consciousness of children returned to normal, speaking speed and action were as usual. She had a history of convulsions. Each convulsion is manifested as loss of consciousness, backward movement of the head, teeth closed, limbs stiff and shaking, no second incontinence, lasting for about 1 min, and can be relieved by itself. She was tired and went to sleep after convulsion. During the month prior to admission, the child developed frequent eyelid blinking and decreased consciousness following frequent eye closure. Combined with the patient's clinical manifestations, EEG, closed eye sensitivity, photosensitivity, and history, we diagnosed the patient as with eyelid myoclonic status. She has no positive family history.

After admission, the biochemical examination was completed. There was no significant abnormality on her biochemical examination, and no abnormality on her head MRI. When we arranged the IQ test for the patient, the family members thought that the daughter did not show cognitive impairment with normal life, and no mental decline was found. So the IQ test was refused. After being diagnosed, the girl was given sodium valproate 1.5 ml bid orally, and the patient responded well to valproic acid, without obvious side effects. On the 8th day of admission, the child had no convulsions and was discharged. In addition, we recommended to the patient to check the liver and kidney function, blood routine, and blood drug concentration detection regularly, drug increase or decrease according to clinical manifestations, and EEG examination. Moreover, mental tests should be performed to determine whether the cognitive aspects of the patient are affected. She was also told to watch for if other types of epilepsies occur and avoid light stimulation. The patient was followed-up by a call for 2 years. She had no recurrence. The girl is now in junior high school and has no obvious adverse reactions or cognitive changes.

## Discussion

Eyelid myoclonus with or without absences (EMA, Jeavons syndrome) has episodic EEG that is typically characterized by extensive high-amplitude polyspike slow-wave discharges of 3–6 Hz lasting for 1.5–6 s. Epileptic discharges occur during or before eyelid myoclonus. Most studies have shown that Jeavons syndrome has eye closure sensitivity (ECS) and photosensitivity. The characteristic epileptic discharge appears within 1–3 s after eye closure and lasts 1–4 s. However, the discharge does not last for the whole time after eye closure. This is a called ECS, and it is different from fixation-off sensitivity (FOS). In FOS, the epileptic discharges persist for the whole duration of eye closure and disappear till eye opening (11). In this case, the abnormal discharges persisted and became more pronounced after eye closure. Vaudano et al. (12) conducted a detailed study of the eyelid myoclonus visual system and found that patients with EMA had abnormal changes at the anatomical and functional level. In patients with EMA, the gray matter content in bilateral peripheral calcarine cortex increased, demonstrating hyperexcitability of the primary visual cortex and impaired intracortical inhibition in photosensitive patients (13). The increased activity of the posterior thalamus and dysfunction of the occipital cortex are involved in the pathogenesis of ECS (14). In patients with EMA, hemodynamic demands of the parietal cortex increase during eye movement, especially around the intraparietal sulcus, which is a network structure that participates in the photoparoxysmal response (PPR) (15). Combined with this case, the patient had eyelid myoclonus status accompanied by continuous blinking or eye closing, so the ECS phenomenon exists in continuous discharge and was covered by a mass of abnormal discharges. We speculated that the frequent blinking action was both manifestation and triggering factors in myoclonic eyelid status.

The eyelid myoclonus patients are photosensitive. That is a characteristic, an abnormal EEG change called photoparoxysmal response, and occurs in about 75% of patients. It can cause episodic symptoms (10). Prolonged IPS stimulation may induce generalized seizures, so patients with a lower IPS onset threshold should be closely monitored. Patients may have one or more IPS-related seizures, such as juvenile myoclonic epilepsy (JME), absence seizures, childhood epilepsy with occipital paroxysms, generalized tonic–clonic seizures, etc. However, routine IPS may not be sufficient to provoke photosensitive seizures. Therefore, the positive rate can be increased by sleep deprivation or prolonged IPS (16). Siniatchkin et al. (17) demonstrated that PPR is associated with increased excitability in the occipital cortex. The increased excitability in the occipital cortex may explain the abnormal response of the occipital cortex to visual input, including super excitation of the occipital cortex during IPS. Surprisingly, after the patient had 2, 4, 6, 8, 10, 12, 14, and 16-Hz IPS, her seizures and epileptic discharges reduced or stopped. We had three extrapolations: First, the frequent blinking action was both a manifestation of eyelid myoclonic seizures and a triggering factor, continuous blinking action led to repeated eye closing movements, resulting in continuous epileptic discharge. When performing IPS, the patients kept their eyes open (we ask patients to keep their eyes open as much as possible), so the blinking movement, epileptic discharges, and seizures reduced. This was consistent with the report of Dede et al. (18). Moreover, a previous study suggested that the eye closure mechanism may be a more potent precipitating factor than photosensitivity in the pathophysiology of EMA (8). Second, because of the neuronal exhaustion during SE, the patient could not respond to IPS. Third, it is just a coincidence because there are natural fluctuations during SE. Therefore, further investigation of the eyelid myoclonic status is urgently needed.

As the intensity of the IPS increases, the cortical excitability of the patient increases, leading to increased epileptic discharges. Just like this case, the patient had eye blinking movement again after 16 Hz and besides, 7 min. After given the stimulation at 20 Hz, the children developed an occipital initiated tonic–clonic seizures called photo-convulsive responses. It demonstrated that after sufficient IPS stimulation, the occiput cortex became excited and initiated a brain network, including the thalamic–cortex and brainstem, leading to diffuse brain discharge and tonic–clonic seizures (5, 19). So the discovery of focal discharges interictal and ictal posterior in eyelid myoclonus patients had led to the hypothesis that EMA was a generalized epilepsy induced by the occipital cortex (4). This hypothesis was validated by Dr. Lee's report of a patient with eyelid myoclonus who developed occipital tonic–clonic seizures following photic stimulation (19). Many epilepsies or epileptic syndromes have a visual aura. Gungor-Tuncer et al. had observed 11 cases of IGE patients with visual aura, including absence of seizures, eyelid myoclonia, eyelid myoclonia with absences, juvenile absence epilepsy, juvenile myoclonic epilepsy, and visual reflex seizures. One of the patients showed up with occipital-originating ictal after IPS (5). Viravan et al. had found two electroencephalographic features in EMA patients in their study; in males, there were predominant epileptic discharges in the frontal, and the clinical manifestations were eyelid myoclonia, and the eyes were rolling up. In the female group, there were predominant epileptic discharges in the occipital with eyelid myoclonia alone, but their eyelid myoclonia is associated with eyes rolling up or absences when their epileptic discharges spread to involve the frontal lobe. However, both the frontal and occipital lobes could trigger generalized discharges and generalized epileptic seizures through either transcortical or thalamocortical pathways (20). Taylor et al. had reported the coexistence of patients with idiopathic generalized epilepsies and photosensitive occipital lobe epilepsy in the same family, and also reported electroclinical features that suggest overlap between idiopathic generalized epilepsies and photosensitive occipital lobe epilepsy. these diseases share common genetic determinants and complex genetic characteristics. the coexistence of generalized and focal seizures within families suggests that rigid clinical distinctions between these groups may not hold true (21, 22).

After patients had a seizure onset, the eyelid myoclonus status symptoms disappeared, the epileptic discharge on EEG also decreased significantly, and even disappeared completely. However, 30 min after onset, the patient's EEG showed epileptic discharge again and gradually increased to the initial state-continuous discharge (1 hour after onset) but the patient's clinical symptom is not obvious with eyelid jerk, but patient drowsiness and consciousness impairment, indicating that the patient was experiencing a non-convulsive status. So we gave the patient midazolam intravenous. The discharge showed in EEG improved significantly after 3 min and completely disappeared after 10 min. The patient had a complete recovery of symptoms, normal communication, normal behavior, and activities. Epileptic status (SE) is a condition resulting from the failure of the mechanisms responsible for seizure termination or the initiation of mechanisms, leading to abnormally prolonged seizures defined by the International Anti-Epilepsy Alliance (23). In other words, it is caused by excessive excitement or the loss of endogenous inhibition mechanisms during seizures. These changes led to the transformation of a single epileptic seizure into the epileptic status and drug resistance (24). Two operational time points, T1 and T2, for epileptic status are proposed. T1 represents the time to start treatment, and T2 represents the time when adverse outcomes (including neuronal death, neuronal damage, and changes in neuronal networks) are likely to occur. Based on preclinical and clinical studies, these two time points are 5 min for T1 and 30 min for T2 (23). SE is a neurological emergency requiring urgent antiepileptic therapy and rapid treatment. Benzodiazepines are the first choice for SE, then antiepileptic drugs and even anesthetics. The prognosis depends on the etiology, age, and course of the disease. Therefore, the patient should be carefully evaluated, and an appropriate treatment plan should selected (25). Apparently, this patient had a significant response to midazolam. She had obvious relief within 3 min after medication. The etiology of ES includes symptoms such as stroke, intoxication, malaria, encephalitis, etc., in the acute phase; posttraumatic, postencephalitic, poststroke, etc., in the remote phase; and brain tumor, Lafora's disease, dementias, neurodegenerative, metabolic, neurogenetic, malignant and neurocutaneous disease in the progressive phase. Idiopathic/cryptogenic SE is referred to as status epilepticus in a patient without a clear cause (23, 26).

Clinically, eyelid myoclonus with absence has some similarities with typical absence epilepsy. Absence epilepsy may also be accompanied by rhythmic eyelid twitching, but the blinking is not the first or most crucial movement, and the EEG in the seizure phase presents as a 3-Hz spike–slow wave, which flares up and stops with normal background activity (27). In typical absence epilepsy patients, absence seizures last longer than eyelid myoclonus and have a more severe impairment of consciousness. Eyelid myoclonus seizures with or without absence are often produced by eye closing or photo stimulation. In addition, the triggering neural network of the two seizures are different. Studies had shown that in absence patients, the epileptic discharges start in the thalamus before the cortex, and the triggering area was in a specific region of the thalamic–cortical system (28, 29). In EMA, PPR is produced from the occipital cortex or frontal cortex (12). Eye closure and IPS can activate the occipital cortex and spread the excitabilities to the brainstem to produce EM (4). However, recent studies have shown that absence epilepsy in children may be transformed into eyelid myoclonic epilepsy, and it is speculated that the sensitivity changes of brain networks in epilepsy patients have a specific time cycle, such as childhood and adolescence, and epilepsy syndrome can evolve dynamically with changes in age and different sensitivities. For example, absence epilepsy can evolve into juvenile myoclonic epilepsy, and eyelid myoclonic epilepsy can also evolve into juvenile myoclonic epilepsy (30).

Eyelid myoclonus with or without absence should be differentiated from other idiopathic, cryptogenic, or symptomatic epilepsy (12). Sunflower syndrome is a reflex seizure. The main clinical manifestations are typically eyelid myoclonia with or without absence seizures triggered when patients wave their hands in front of the sun. Eyelid myoclonus is a genetic disorder. Mutations or deletions of related genes have been reported in eyelid myoclonus, such as RORB gene (31), KIAA2022 gene (32), GLUT1 gene (33), and NAA10 gene (34). Therefore, the pathogenic genes can be confirmed by genetic testing. Some photosensitive IGE needs to be differentiated from EMA, such as juvenile myoclonic epilepsy (JME). JME is a heterogenetic generalized epilepsy (GGE) syndrome that begins at puberty and characterized by myoclonic convulsions, mainly in the upper extremities. It can be accompanied by tonic-clonic seizures (TCS), absence, PPR, ECS, and EM. Myoclonus occurs after frequent awakening from sleep (35). The EEG is more likely to detect abnormalities in the morning, and sleep EEG always shows epileptic discharges (11). Idiopathic photosensitive occipital lobe epilepsy is a rare but well-defined syndrome in the group of idiopathic focal epilepsies. It is characterized by changes in vision, including hallucinations, complex hallucinations, blindness, or both. Simple vision is common (two thirds of cases); eyelid closure and blinking, secondary generalized tonic–clonic seizures (GTCs), and complex focal seizures have also been reported in patients with idiopathic photosensitive occipital lobe epilepsy. Intermittent luminous stimulation can also trigger epileptic seizures. Unlike EMA, interictal EEG showed that occipital paroxysms occur when the eyes are closed, and disappear or attenuated when eyes are opened. Therefore, electroencephalogram is a good test to distinguish between EMA and idiopathic photosensitive occipital lobe epilepsy (36).

Valproic acid monotherapy is the most effective treatment for eyelid myoclonus with or without absence, which should be considered early. Patients who cannot tolerate valproic acid, lamotrigine, or a combination should be considered. Previous studies had shown that levetiracetam monotherapy, even at high doses, was unlikely to be effective (37). Verrotti et al. first proved that VPA monotherapy was very effective for both seizure outcome and photosensitive (PS) reduction or abolition in adolescents with photosensitive epilepsy with generalized tonic–clonic seizures (EGTCS). In their study, seizures disappeared in 69.1% of patients, and PS disappeared in 52.7% of patients after 6 months of treatment. During valproic acid treatment, seizures were more easily controlled than PS, but the potential of epileptic discharge could be revealed by IPS. Consequently, they argued that control of PPR in EGTCS patients might be a prognostic factor for better seizure control. VPA therapy may be more effective in generalized seizures than PPR, since PPR is the initial point of occipital discharge (38). A recent retrospective analysis of GGE patients also showed that valproic acid effectively reduced spike–slow waves induced by spontaneous and hyperventilation, but not in inhibiting spike–slow wave induced by IPS (9). It has also been mentioned that EM was an age-related electrical clinical manifestation that tends to disappear between 15 and 18 years old, even if the patient previously had only a partial response to different treatments (35). Studies have shown that photosensitivity, ECS, and family history can be used as prognostic indicators of eyelid myoclonus. The persistence of photosensitivity and ECS indicates the possibility of an epileptic seizure, and positive family history is a benign indicator (6). Therefore, it demonstrates the importance of EEG in the diagnosis and prognosis evaluation of EMS.

Patients with eyelid myoclonic seizures are often getting the attention of the patient or family for generalized tonic–clonic seizures, which usually occur at age 12. It also proves that the early diagnosis of Jeavons can be easily missed or even misdiagnosed, leading to the wrong antiepileptic, such as carbamazepine, which could actually exacerbate the disease (24). Like the girl in this case, she had a history of four convulsions in the 1.5 years before this visit. However, the patient's family did not follow the doctor's advice for a systematic examination, and thus, no diagnosis was given, which eventually led to the patient's epileptic status. Therefore, the enlightenment to us is that doctors should actively educate, encourage, and guide patients and their families to actively face the disease because early recognition of the disease has a significant impact on the prognosis and treatment.

## Conclusion

Eyelid myoclonus with or without absence epilepsy is a relatively rare type of epilepsy in clinical practice. It is necessary to enhance doctors' awareness about this disease and improve the detection rate. The occurrence of eyelid myoclonus status is rarer, and it is rarely reported.

## Data Availability Statement

The original contributions presented in the study are included in the article/supplementary material further inquiries can be directed to the corresponding authors.

## Ethics Statement

Ethical review and approval was not required for the study on human participants in accordance with the local legislation and institutional requirements. Written informed consent to participate in this study was provided by the participants' legal guardian/next of kin.

## Author Contributions

YY and HW designed and guided the work. YY collected the case and drafted the article. FY and YF collected and analyzed the case. LH and QW analyzed and followed-up the case. XL guided interpretation and revision of manuscript. All authors contributed to the article and approved the submitted version.

## Conflict of Interest

The authors declare that the research was conducted in the absence of any commercial or financial relationships that could be construed as a potential conflict of interest.
